# Monolithic carbon structures including suspended single nanowires and nanomeshes as a sensor platform

**DOI:** 10.1186/1556-276X-8-492

**Published:** 2013-11-20

**Authors:** Yeongjin Lim, Jeong-Il Heo, Marc Madou, Heungjoo Shin

**Affiliations:** 1 School of Mechanical and Advanced Materials Engineering, Ulsan National Institute of Science and Technology (UNIST), Ulsan 689-798, Republic of Korea; 2 School of Nano-Bioscience and Chemical Engineering, Ulsan National Institute of Science and Technology (UNIST), Ulsan 689-798, Republic of Korea; 3 Department of Mechanical and Aerospace Engineering, University of California-Irvine, Irvine, CA 92697, USA

**Keywords:** Suspended carbon nanostructure, Pyrolysis, C-MEMS, Nanomesh

## Abstract

With the development of nanomaterial-based nanodevices, it became inevitable to develop cost-effective and simple nanofabrication technologies enabling the formation of nanomaterial assembly in a controllable manner. Herein, we present suspended monolithic carbon single nanowires and nanomeshes bridging two bulk carbon posts, fabricated in a designed manner using two successive UV exposure steps and a single pyrolysis step. The pyrolysis step is accompanied with a significant volume reduction, resulting in the shrinkage of micro-sized photoresist structures into nanoscale carbon structures. Even with the significant elongation of the suspended carbon nanowire induced by the volume reduction of the bulk carbon posts, the resultant tensional stress along the nanowire is not significant but grows along the wire thickness; this tensional stress gradient and the bent supports of the bridge-like carbon nanowire enhance structural robustness and alleviate the stiction problem that suspended nanostructures frequently experience. The feasibility of the suspended carbon nanostructures as a sensor platform was demonstrated by testing its electrochemical behavior, conductivity-temperature relationship, and hydrogen gas sensing capability.

## Background

The advantageous physicochemical properties of many of the different carbon microstructures have attracted a wide range of research interests and a large variety of carbon allotropes ranging from graphene sheets to carbon nanotubes (CNTs), diamond-like coatings, and glassy carbon have been investigated intensively [1-4]. Glassy carbon is one of the carbon allotropes of particular interest in this study; it exhibits a wide electrochemical stability window, excellent biocompatibility, superior thermal and chemical stability, low gas permeability, and high thermal conductivity [5]. The low reactivity and gas impermeability of glassy carbon has been explained by a fullerene-related model that holds that glassy carbon contains primarily non-graphitizing *sp*^2^-bonded carbons [6]. Glassy carbon has been explored for applications in solar cell systems [7], Li-ion batteries [8], optical memory devices [9], and electrochemical sensing platforms [10].

To enable these listed applications, several research groups are working towards low-cost carbon fabrication processes. Interesting three-dimensional (3D) glassy carbon shapes can often be obtained simply by patterning certain polymer precursors into the desired geometry and heating it at high temperature in an inert atmosphere or in vacuum, i.e., by pyrolysis or carbonization [11]. Based on this general fabrication scheme, various types of polymer patterning processes and pyrolysis process variations are combined to extend the applications of glassy carbon devices. Polyfurfuryl alcohol (PFA) [12-14] and photosensitive polymers [5,10,15,16] are widely used as polymeric precursors for glassy carbon. Glassy carbon nanowires were fabricated, for example, by the pyrolysis of poly furfuryl alcohol nanowires polymerized in the pores of a nanoporous alumina template and subsequent template removal [13]. These nanowires exhibited semiconductor-type electrical properties as also found in semiconducting amorphous materials [17]. However, with a technique like this, it is difficult to position carbon nanowires at desired locations of pre-existing structures for the completion of micro/nanodevices or for realizing reliable ohmic contacts with the nanowire at desired points along the nanowires. A more versatile fabrication method called carbon microelectromechanical systems (C-MEMS) was developed; it is capable of generating monolithic 3D carbon micro/nanostructures, inclusive of ohmic contacts, by pyrolyzing photosensitive micro/nanopolymer structures pre-patterned using any type of lithography including UV lithography and e-beam lithography [8,16]. Especially when UV lithography is used to pattern the polymer structures, C-MEMS constitutes a simple and relatively low-cost fabrication method [5,10,15]. During pyrolysis, the polymer precursor experiences dramatic volume shrinkage and that shrinkage is isometric and predictable. The smaller the original polymer feature size, the more dramatic the shrinkage, and micrometer-sized features shrink as much as 85% to 90% [10]. In an interesting variation on this process, suspended carbon nanowires between walls and posts were fabricated using a combination of UV lithography and electrospinning [18]. The electrospun nanowires were pyrolyzed together with the UV lithographically patterned SU-8 photoresist ensuring good ohmic contact between walls/posts and wires [19,20]. The reason these authors wanted to fabricate suspended carbon nanowires was to avoid deleterious substrate effects and to enhance mass transport in gases and liquids to the sensing element.

In the current study, we prepared monolithic suspended carbon nanostructures, including nanowires and nanomeshes, which were patterned by two successive UV exposure processes and a single pyrolysis process. The microstructure of the carbon nanowire and the development of stress along the wire were explored using a focused ion beam (FIB) milling process, scanning electron microscopy (SEM), and high-resolution transmission electron microscopy (HRTEM). The intrinsic tensile stress along the nanowire and its bent supports mitigated stiction problem and this structural advantage was explored by executing photolithography, metal deposition, wet etching, and electrochemical experiment on an approximately 200-nm-diameter suspended carbon nanowire. In order to confirm the feasibility of suspended carbon nanostructures as nanosensors, their electrical, electrochemical, and thermal properties were characterized experimentally and through simulations. Moreover, the carbon nanowire was selectively coated with palladium using a lift-off process and its functionality as a hydrogen gas sensor was tested.

## Methods

**Figure 1:**
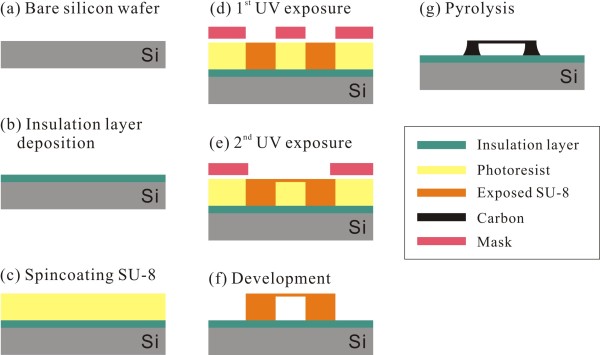
Schematic fabrication steps of suspended carbon nanostructures. **(a)** A bare silicon wafer, **(b)** insulation layer deposition, **(c)** spincoating SU-8, **(d)** UV exposure for carbon posts, **(e)** UV exposure for suspended carbon structures, **(f)** development, **(g)** pyrolysis.

The schematic fabrication steps of the suspended carbon nanostructures are described in Figure 1. First, a 0.5-μm-thick SiO_2_ layer was grown on a 6-inch Si wafer (p-type, boron doped, 8 to 12 Ω · cm^2^, 660-μm thick) using thermal oxidation. The SiO_2_/Si substrate was cleaned in a hot piranha solution (H_2_SO_4_/H_2_O_2_ = 4:1) and dehydrated on a hot plate at 200°C for 5 min. After a 35-μm-thick layer of negative photoresist (SU-8 2000, MicroChem, Corp., Newton, MA, USA) was spin-coated onto the SiO_2_/Si substrate and soft-baked at 95°C for 9 min, a long UV exposure (200 mJ · cm^−2^) was performed through a photomask defining post structures. A second UV exposure with lower dose (22 mJ · cm^−2^) was subsequently performed to polymerize only the shallow area of the negative photoresist layer. The UV lithography process was finished by a post-exposure bake (95°C for 8 min) and a development step. Finally, the photoresist structures consisting of posts and suspended photoresist mircrowires were pyrolyzed in a vacuum furnace and converted into monolithic carbon structures. The pyrolysis process consisted of a pre-baking step for degassing and major volume reduction and a carbonization step for forming solid carbon. During pre-baking, the suspended photoresist microsized wire structures were heated to 350°C at a rate of 1°C/min and maintained at that temperature for 60 min. The temperature was then increased to 900°C at a rate of 1°C/min and maintained at that temperature for 60 min. Finally, the wafer was steadily cooled to the room temperature.

The shape and microstructure of the suspended carbon nanostructures were characterized using a SEM (Quanta 200, FEI company, Hillsboro, OR, USA), a HRTEM (JEM-2100 F, JEOL Ltd., Tokyo, Japan), a FIB (Quanta 3D FEG, FEI company, Hillsboro, OR, USA), and a Raman spectroscopy systems (alpha 300R, WITec GmbH, Ulm, Germany). The crystallinity of the pyrolyzed carbon was analyzed by comparing the HRTEM diffraction patterns of a suspended nanowire and the Raman spectroscopy results of bulk carbon structures. The change in the composition of the SU-8 structures after pyrolysis was confirmed using XPS (K-Alpha, Thermo Fisher Scientific Inc., Waltham, MA, USA).

The temperature-dependent resistivity change was recorded using a Keithley 2400 SourceMeter (Keithley Instruments Inc., Cleveland, OH, USA) while varying the temperature of the suspended carbon nanowire in a natural-convection oven (ON-02GW, JEIO TECH CO., Ltd., Seoul, South Korea). The samples were equilibrated for 2,000 s at each temperature to ensure that the temperature of the carbon nanowire coincided with the oven temperature. The applied current value was limited to ≤10 μA to avoid nanowire temperature increase due to Joule heating.

Electrochemical properties were established using a multichannel potentiostat (CHI-1020, CH Instruments, Inc., Austin, TX, USA) for recording cyclic voltammograms of single suspended carbon nanowires in a 10 mM ferricyanide (Sigma-Aldrich Co. LLC., St. Louis, MO, USA) and 0.5 M KCl (BioShop Canada Inc., Burlington, ON, Canada) solution. The voltage was scanned from 0.6 V to −0.2 V at a ramp rate of 0.05 V · s^−1^ against an Ag/AgCl reference electrode, and a Pt wire was used as a counter electrode. Diffusion-limited currents from a suspended carbon nanowire and a non-suspended wire (planar on a solid substrate) were calculated and compared to each other using COMSOL Multiphysics (ver. 4.2a, COMSOL, Stockholm, Sweden) software to confirm the effects of geometry of the suspended structures on the electrochemical current signal.

The feasibility of a single suspended carbon nanowire as a hydrogen gas sensor was tested by surface functionalization with palladium. A single carbon nanowire was coated with a 5-nm-thick palladium layer using an e-beam evaporation process subsequent to a photolithography process in which only a suspended region of the carbon structure was exposed in the metallization process. After metal deposition, the photoresist layer was stripped off using a wet process. The resistance change of the palladium-coated carbon nanowire in response to the concentration change of hydrogen gas mixed with air was recorded.

## Results and discussion

**Figure 2:**
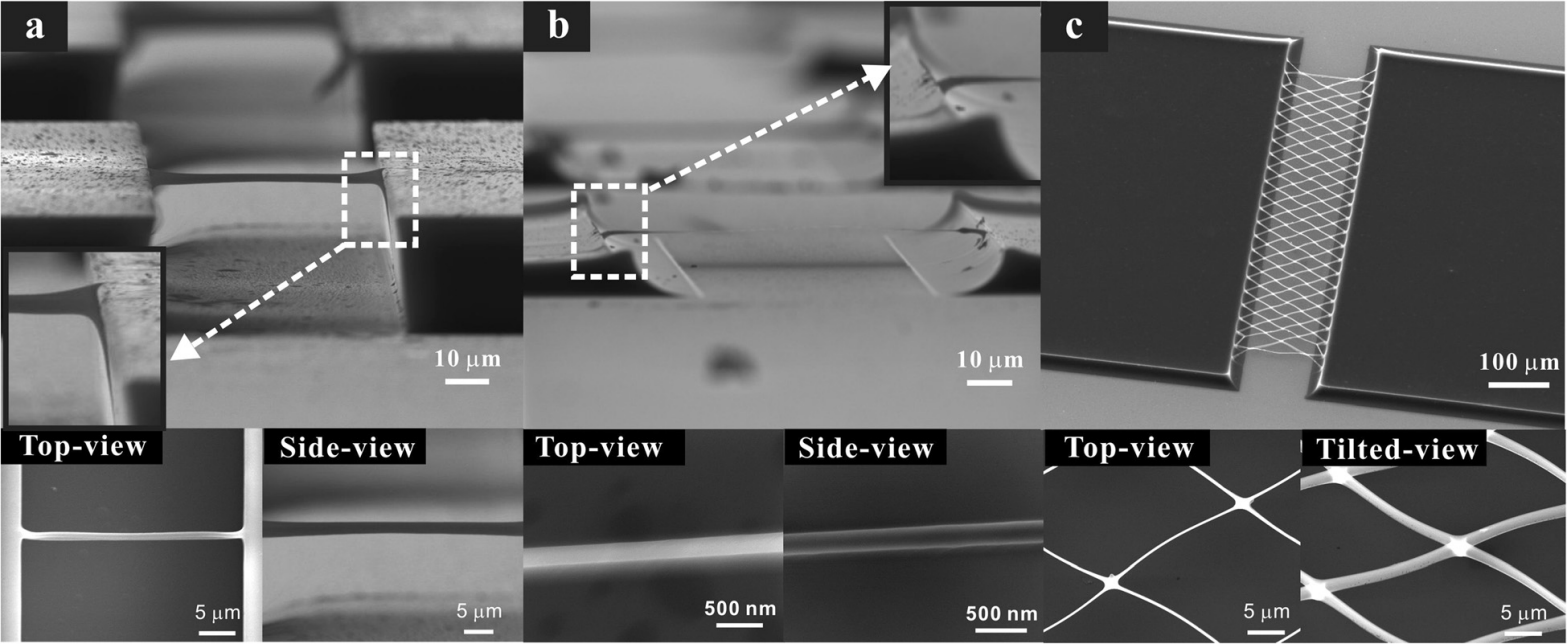
**SEM images of suspended SU-8 microwire structure, a corresponding carbon nanowire structure, and suspended carbon nanomesh. (a)** A suspended SU-8 microwire structure before pyrolysis and **(b)** a corresponding suspended carbon nanowire structure after pyrolysis. **(c)** A suspended carbon nanomesh. Inset images of **(a)** and **(b)** are the enlarged views of the polymer and carbon supports.

Formation of suspended carbon nanostructure of predefined shapes and locations was realized by combining UV lithography and pyrolysis. The shape of the carbon nanostructures bridging the two carbon posts is roughly an isometrically shrunk version of the suspended SU-8 photoresist microsized structures connecting the two SU-8 posts, as shown in Figure 2a,b. The width of the photoresist wire coincided with the photomask pattern size but the polymer wire thickness varied depending on the total UV light absorbed by the photoresist as determined by the second UV exposure. For the same pyrolysis duration, polymer structures experience different amounts of shrinkage ranging from 40% to 90% depending on the original polymer structure sizes, as listed in Additional file 1: Table S1 of the Supporting Information. The smallest polymer microwire that was 1-μm wide and 2-μm thick was converted to a carbon nanowire 195-nm wide and 210-nm thick, corresponding to 80% to 90% size reduction. On the other hand, the length of the carbon nanowire increased from 54.0 to 89.4 μm due to the volume shrinkage of the two posts supporting the wire. Even with this large elongation (65.6%), the resulting longitudinal tension in the carbon nanowire was not significant, as demonstrated in an FIB milling experiment of the carbon nanowire (Supporting Information Additional file 1: Figure S1). We found that the sum of the lengths of two FIB sectioned carbon nanowires was not significantly different from that of a single carbon nanowire before sectioning; this means that the carbon nanowire does not have much tensional stress (in which case, we would have expected the wires to ‘spring back’). Importantly, the carbon nanowires were slightly bent upwards. We believe that these points towards the development of a transverse gradient of stress along the nanowire thickness, that is the top part of the nanowire is under more tensional stress than the bottom part of the nanowire when the nanowire is not sectioned. From this result and from experiments on the amount of volume shrinkage as a function of the pyrolysis temperature as listed in the Supporting Information Additional file 1: Table S2, it is deduced that most of the volume reduction of the SU-8 polymer occurs in the early stages of the pyrolysis process, i.e., at temperatures up to approximately 450°C. This is before solid carbon formation takes place as known in the literature [21,22] and where the polymer structure is still sufficiently flexible to endure the large amount of elongation without fracture. The volume keeps decreasing even during the solid carbonization step at temperatures above 450°C to 900°C but the volume shrinkage rate is much less than that in the lower temperature volume reduction step. Therefore, the small amount of longitudinal stress along the carbon nanowire can be explained by the fact that most of the dimensional changes occur in the polymer phase and only small dimensional changes occur during the solid carbon formation itself. It also should be stressed that the slow temperature ramp rate of 1°C/min during the pyrolysis process and the slow cooling process afterwards would tend to anneal out any excessive stresses accumulated in the carbon structure. The shape of the supporting posts was converted from a brick shape to a four-pole tent shape and the wire bent downwards at supports where the nanowire and the post are connected as shown in the inset image of Figure 2b and Additional file 1: Figure S2. This geometric shape is a result of the very good adhesion of SU-8 to the substrate, where the bottom part of the posts, during pyrolysis, is held strongly by the substrate while the top of the posts tend to shrink freely inwards and downwards. As a result of this type of non-uniform volume reduction of the posts, the side-wall profile of the posts changes from a straight wall to a curved one and as a consequence the suspended nanowires experience more elongation at the top compared to the bottom and the nanowire supports are bent downwards. It is this difference in the top to bottom elongation across the nanowire thickness that causes the transverse stress gradient in the nanowire. The photoresist wires are formed thicker at the supports as shown in the dashed rectangle of Figure 2a because the photomask open area in the 2nd UV lithography process is enlarged abruptly at the supports such that the UV energy is transferred deeper at the ends of the nanowire. The polymer supports remain thicker compared to the wire through pyrolysis and transforms into thick carbon bent supports. This bridge-shaped carbon nanowire geometry and the tensional stress, that is not significant but grows along the nanowire thickness, enhanced the structural robustness of the nanowire and could enable high aspect ratio (approximately 450) suspended carbon nanowires to resist stiction to the substrate even when they were wet processed with very small gaps between the nanowires and the substrate.

**Figure 3:**
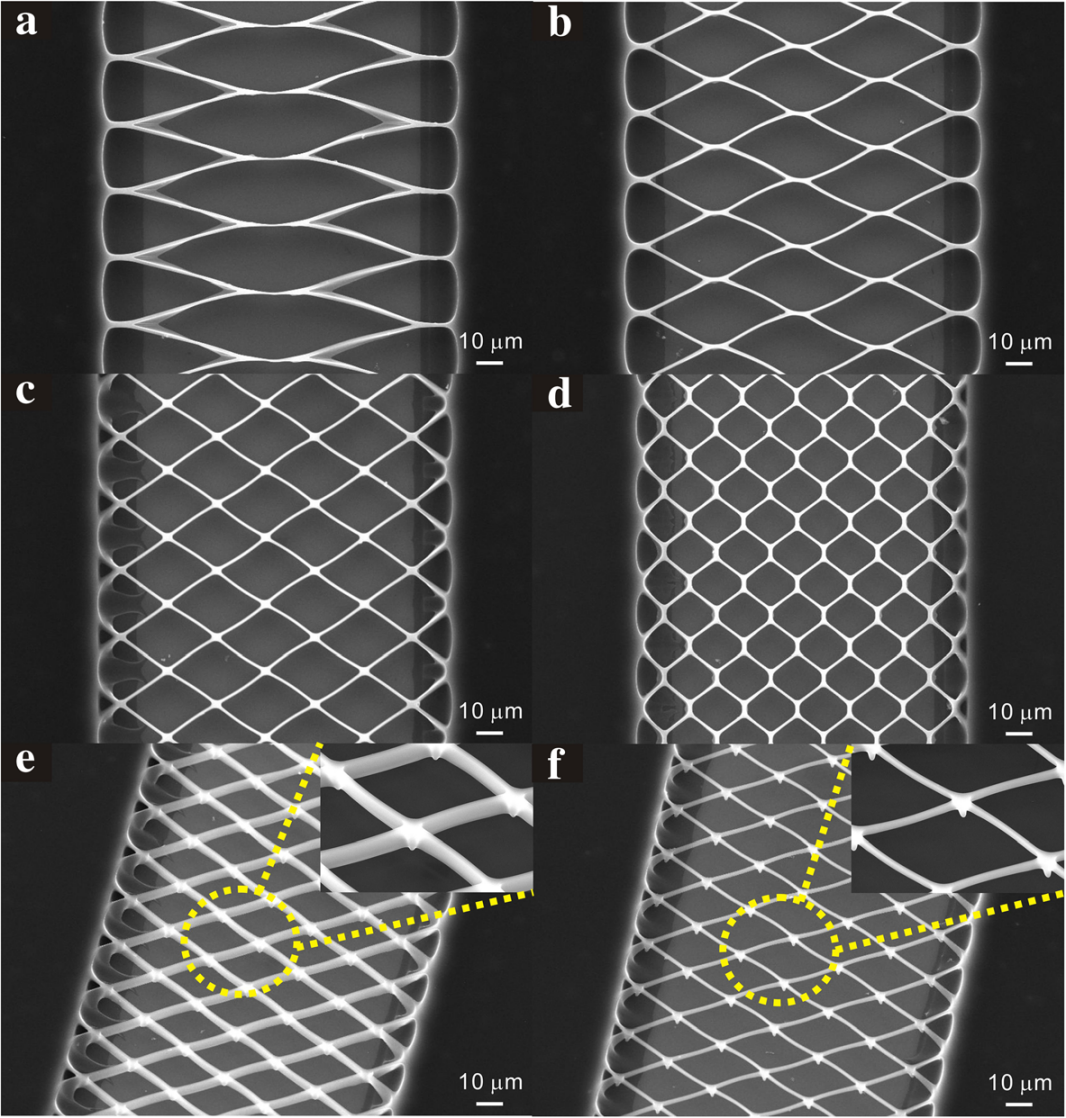
**Scanning electron microscopy images of various types of suspended carbon nanomeshes. (a)** A football-shape, **(b,c)** diamond shapes, **(d)** a hexagonal shape, **(e)** a vertically erected nanobelt type, **(f)** a nanowire type.

In contrast to suspended carbon nanowires fabricated using electrospinning, the UV lithography-patterned suspended carbon nanowires can be shaped in a wide variety of geometries such as nanomeshes. Dimensions including width and aspect ratio, and locations of SU-8 precursor structures are determined by the photomask patterns and the UV dose as shown in Figure 3. The resultant nanomesh sectional geometries varied from vertically erected nanobelts or nanowires depending on the size of the photomask patterns and the UV dose in the second photolithography process as shown in Figure 3e,f. The suspended carbon nanomeshes are designed to align obliquely to the bulk carbon post edges so that each junction, where four short carbon nanowires intersect, is supported evenly by the four nanowires. This robust mesh design avoids stiction between neighboring wires due to surface tension during development and breakage of the mesh structures during pyrolysis, and as a result, the nanowires can be spaced with a small gap.

**Figure 4:**
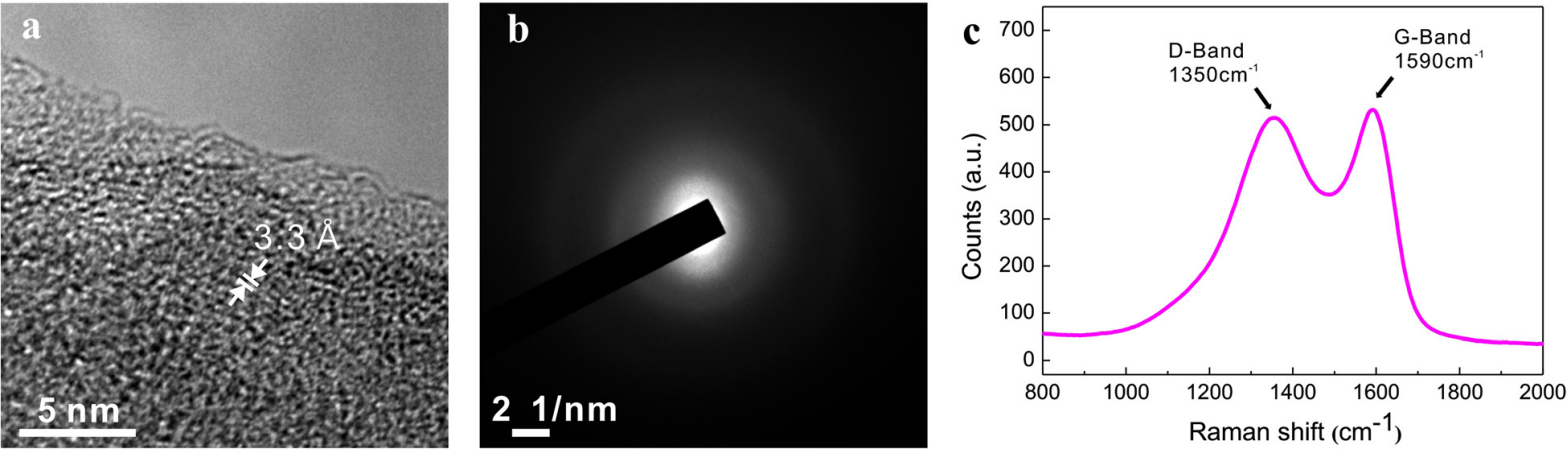
**TEM image (a) and corresponding diffraction patterns (b) of a carbon nanowire and Raman spectrum from a carbon post (c).** The TEM image was obtained at the edge of an approximately 190-nm-size bare carbon nanowire.

The microstructure of the pyrolyzed carbon structures was analyzed using HRTEM and Raman spectroscopy. Figure 4a shows a HRTEM image at the edge of an approximately 190-nm-diameter carbon nanowire. Because the diameter of the suspended carbon nanowire is too large for electrons to be transmitted across the nanowire center, only the edge of a carbon nanowire as-made could be clearly observed in TEM (Figure 4a). The nature of the carbon nanowire is predominantly disordered but shows some short-range ordered nanostructures. The nature of the microstructure of the nanowire was also confirmed by a TEM diffraction pattern, as shown in Figure 4b. The ring shape diffraction pattern indicates a short-range crystalline order, and the foggy pattern surrounded by the ring pattern is indicative of defects in the graphitic phase [23]. This short-range crystalline nature of the pyrolyzed carbon was confirmed by Raman spectroscopy. Due to the limited spatial resolution of the Raman spectroscopy, the carbon post instead of the suspended carbon nanowire was tested as shown in Figure 4c. The G-band at 1,590 cm^−1^ is representative of *sp*^2^ hybridized graphitic material and the D-band shown at 1,350 cm^−1^ stems from disordered carbon [24,25]. The overlapping shape of the D-band and the G-band and the relative intensity of the two bands are consistent with TEM results indicating that the pyrolyzed carbon is a mixture of ordered and disordered carbons.

**Figure 5:**
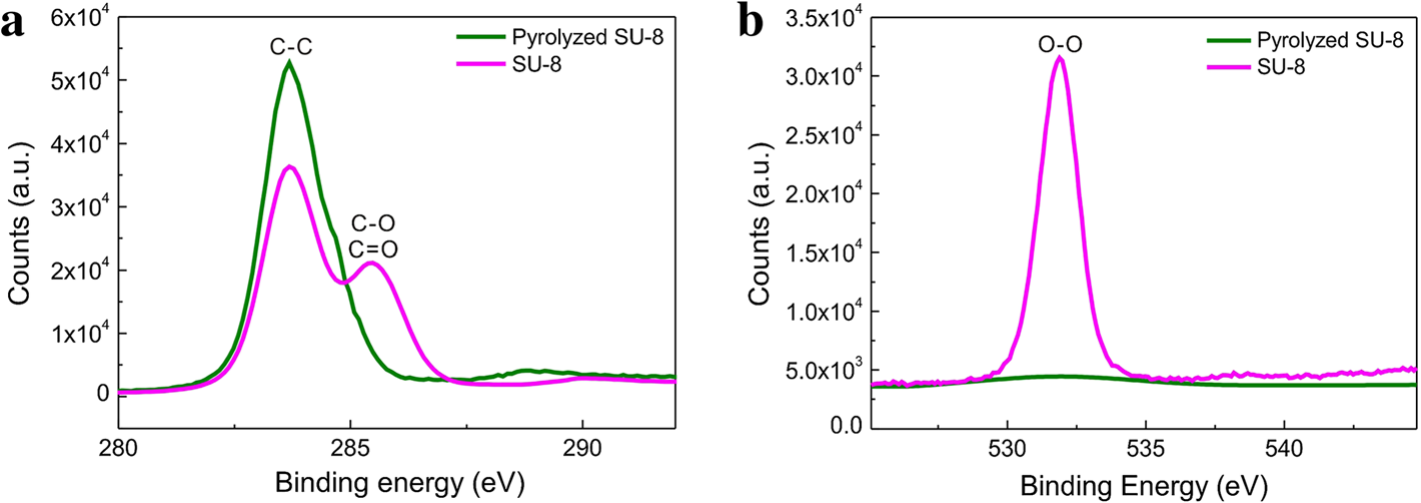
**XPS spectra in (a) C1s and (b) O1s regions.** XPS spectra were obtained from a bare SU-8 structure before pyrolysis and a pyrolyzed bulk carbon structure.

The oxygen-to-carbon (O/C) ratio is often used to characterize the composition of carbonized materials. In Figure 5a,b, we show high-resolution XPS spectra in the C1s and O1s regions, respectively, of a pyrolyzed bulk carbon structure and a SU-8 precursor structure. The C1s spectrum of the SU-8 structure consists of peaks at 283.7 and 285.9 eV. The peak at 285.9 eV corresponds to carbon bound to oxygen and the peak at 283.7 eV represents aromatic and aliphatic carbons in the polymer [26]. The C1s spectrum from the pyrolyzed carbon structure only has a single peak at 283.7 eV. In the O1s spectral region, the pyrolyzed carbon features a peak at 531.8 eV significantly reduced in intensity from the corresponding peak of the SU-8 polymer before pyrolysis. The difference in O/C ratios between the SU-8 polymer structure before (23.2%) and that after pyrolysis (3.1%), confirms a low level of oxygen in the pyrolyzed carbon. This result is in agreement with that obtained on other pyrolyzed carbon structures [27].

**Figure 6:**
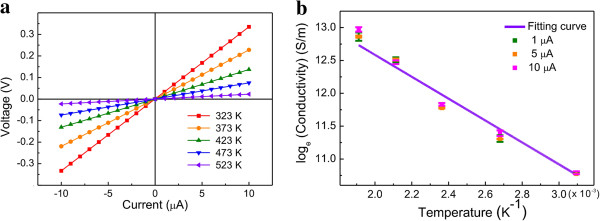
**Conductivity-temperature relationship of a suspended carbon nanowire (size approximately 190 nm). (a)** Voltage versus current curves in various temperature conditions. **(b)** Conductivity to temperature curve in a logarithmic scale.

The electrical properties of the suspended carbon nanowires were evaluated using a two-probe I-V technique using the posts as contact pads instead of using a four-point probe method. A two-probe approach could be used in this case because the effects of contact resistance and spreading resistance, which are the main sources of electric measurement errors, could be neglected here since the nanowire is connected to the post monolithically and the carbon nanowire has a much greater resistance compared to the carbon posts due to their large size difference. Carbon nanowires with a width and thickness of approximately 190 nm showed excellent ohmic contact, and the wire resistance decreased as the temperature increased (Figure 6a). The inverse proportionality of temperature and resistance is indicative of the semiconductor-like behavior of the suspended carbon nanowire. The electrical conduction mechanism in disordered carbon is explained by a hopping-based mechanism at low temperatures (<250 K) [28] and a thermally activated mechanism at higher temperatures (>250 K) [13]. As we made measurements at temperatures above room temperature, the following relationship of conductivity vs. temperature applies [13].

(1) $$\sigma \left(T\right)=\sigma_{0}\exp\left(-\epsilon_{\mathrm{act}}/\left(k_{B}T\right)\right),$$

where *σ*_0_ is a constant, *k*_*B*_ is the Boltzmann constant, and *ϵ*_act_ is the activation energy. The activation energy *ϵ*_act_ is defined as *ϵ*_act_ = *ϵ*_*C*_ − *ϵ*_*F*_, where *ϵ*_*C*_ is the conduction band edge and *ϵ*_*F*_ is the Fermi level. The activation energy obtained by fitting a plot of ln(*σ*) versus *T*^-1^ from the resistance measurement results was approximately 0.146 eV. This small activation energy of the carbon nanowire is also found in predominantly sp^2^ carbonaceous materials such as pyrolyzed polyfurfuryl alcohol nanowires [13] and confirms that the composition of the suspended carbon nanowire is mainly non-graphitizing sp^2^ bonded carbons.

**Figure 7:**
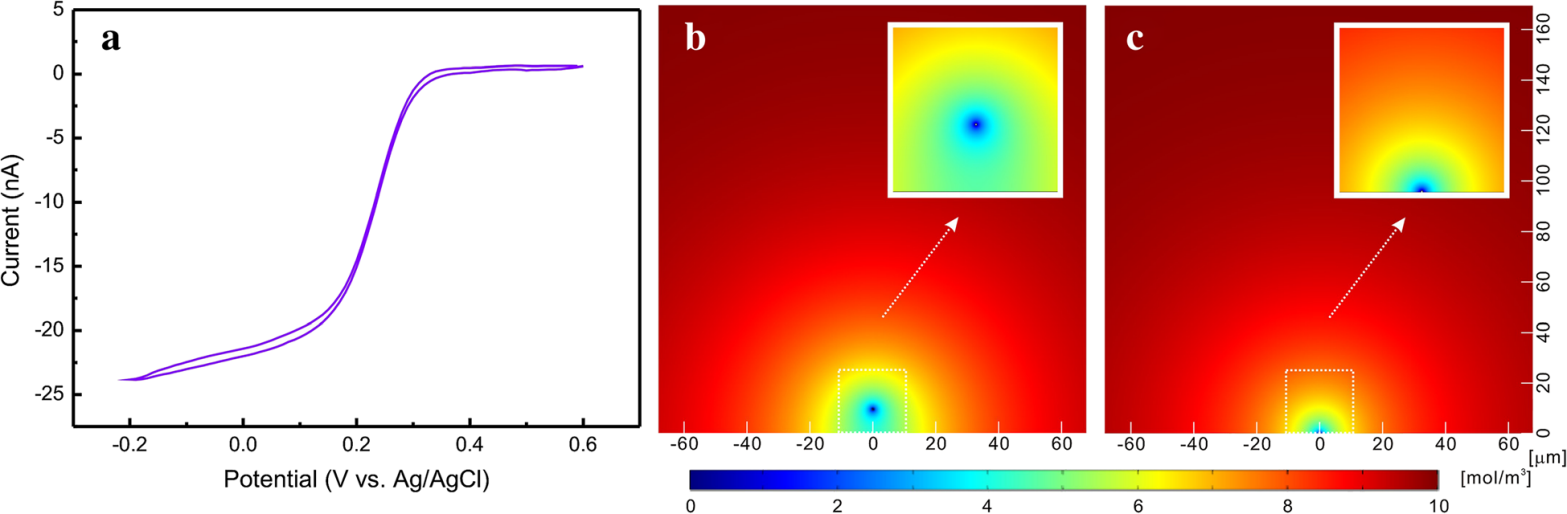
**Cyclic voltammogram of a suspended carbon nanowire (a) and simulated 2-D concentration profiles (b,c). (a)** A cyclic voltammogram was collected from a suspended carbon nanowire (diameter approximately 190 nm) in 10 mM K_3_Fe(CN)_6_ and 0.5 M KCl solution; the monolithic carbon structure was insulated with a negative photoresist pattern except for the 43-μm-long middle section of the nanowire. 2-D concentration profiles were simulated for **(b)** a suspended nanowire and **(c)** a surface-bound nanowire structure with the same section areas as the carbon nanowire used in the cyclic voltammetry as in **(a)**.

The suspended carbon nanowire was characterized electrochemically by cyclic voltammetry in a 10-mM K_3_Fe(CN)_6_ solution with 0.5 M KCl (Figure 7a). The value of the measured diffusion-limited current was compared to the simulated current values from a suspended circular carbon nanowire (Figure 7b) and a surface-bound square carbon nanowire (Figure 7c) with the same effective section areas as the nanowire in our experiment. The detailed simulation procedure is described in the Additional file 1. The measured maximum current at −0.2 V was 23.8 nA, and the simulated results from the suspended nanowire and the surface-bound nanowire were 21.6 and 12.9 nA, respectively. The good agreement between the measured current and the simulated value confirmed that the suspended carbon nanowire surface achieved good electrochemical activity. Only one quarter of the surface area of the surface-bound nanowire was blocked by the substrate surface but the current of the surface-bound carbon nanowire was reduced to 59% of that from the suspended carbon nanowire. This result is indicative of the advantage of the mass transfer of the suspended nanowire structure over the surface-bound nanowire geometry, in addition to the freedom from substrate surface effects such as contamination, substrate temperature change, and delayed response time caused by a stagnant layer.

**Figure 8:**
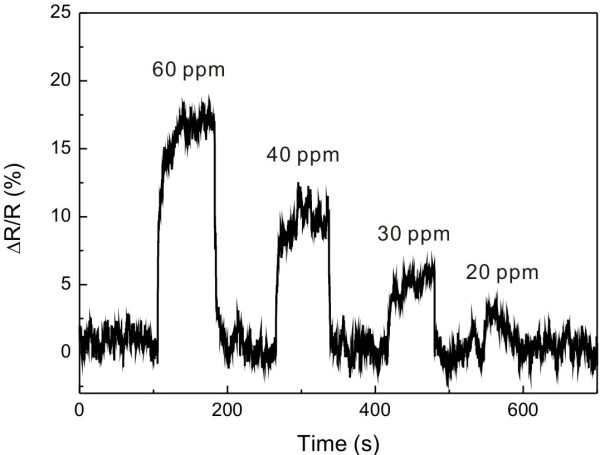
**Hydrogen gas sensing using a suspended carbon nanowire functionalized with palladium.** Resistance change of a suspended carbon nanowire (width = 260 nm, thickness = 380 nm, length = 120 μm) functionalized with a palladium layer (thickness = 5 nm, length = 80 μm) in response to the concentration of hydrogen gas mixed with air was measured.

Palladium is a material of which resistance changes depending on the hydrogen gas concentration so that palladium-based nanostructures are widely used as highly sensitive hydrogen gas sensors [29,30]. In current research, we demonstrated the selective coating of a single suspended carbon nanowire with a thin palladium layer and the gas sensing capability of the functionalized carbon nanowire. A 200-nm-diameter carbon nanowire coated with a 5-nm-thick palladium layer showed distinct resistance change down to 30-ppm hydrogen gas mixed with air as shown in Figure 8. Because of the robustness and suspended geometry of the carbon nanowire, the nanowire could be easily functionalized with sensing materials using a simple lift-off process.

## Conclusions

In summary, we demonstrated the simple batch nanofabrication of monolithic suspended carbon nanostructures, including single nanowires and nanomeshes of scalable dimensions and user-defined designs depending on conditions in UV lithography and pyrolysis. The conversion from the microscale polymer wires to nanoscale carbon wires resulted from volume reduction of negative photoresist structures during pyrolysis under vacuum conditions. The suspended nanowire bridging carbon posts demonstrated perfect ohmic contact due to the monolithic structures. The transverse gradient of the longitudinal tension and the bridge-shaped geometry with thick bent supports of the carbon nanowire ensures high resistance to device failure due to a stiction phenomenon that limits reproducibility and applications of suspended nanostructure-based nanodevices. Furthermore, the overall density of suspended nanowire array could be enhanced by modulating the geometry of the nanowire structures from straight nanowire arrays aligned in parallel to nanomeshes that neither conventional bottom-up nor top-down nanofabrication processes can realize easily. The linked structure of the nanomeshes ensured better structural robustness than that of a linearly aligned nanowire array. We believe that the advantageous properties of the suspended carbon nanostructures including cost-effective batch nanofabrication procedure, semiconductor type electrical conductivity, electrochemical sensing capability, easy surface functionalization, structural robustness, and suspended geometry will enable those nanostructures to work as platforms for a variety of nanodevices such as gas sensors, biosensors, and nanogenerators that can be implemented by simply coating functional materials on the suspended carbon nanostructures.

## Competing interests

The authors declare that they have no competing interests.

## Authors’ contributions

YL carried out the fabrication and characterization of the suspended carbon structures and drafted the manuscript. JIH participated in the fabrication of the suspended carbon structures. MM provided the scientific advice about the experiment. HS supervised the whole study. All authors read and approved the final manuscript.

## Supplementary Material

Additional file 1 **Supporting Information.** The file contains discussion on the longitudinal tension and geometry of suspended carbon nanowires and the simulation of the diffusion-limited current of a suspended carbon nanowire. **Figure S1.** Schematic diagrams and SEM images of FIB milling processes. **Figure S2.** SEM images of bridge-shaped carbon nanowires with bent supports. **Table S1.** Structural dimension change of suspended carbon nanostructures through the pyrolysis process. **Table S2.** Structural dimension changes of suspended SU-8 microwires and bulk posts in various pyrolysis temperature conditions. Click here for file
