# Supplementary material for: Monolithic carbon structures including suspended single nanowires and nanomeshes as a sensor platform
Source: Nanoscale Res Lett. 2013 Nov 20;8(1):492. doi: 10.1186/1556-276X-8-492 (PMC3874666; doi:10.1186/1556-276X-8-492)
Supplement: Additional file 1 — Supporting Information. The file contains discussion on the longitudinal tension and geometry of suspended carbon nanowires and the simulation of the diffusion-limited current of a suspended carbon nanowire. Figure S1. Schematic diagrams and SEM images of FIB milling processes. Figure S2. SEM images of bridge-shaped carbon nanowires with bent supports. Table S1. Structural dimension change of suspended carbon nanostructures through the pyrolysis process. Table S2. Structural dimension changes of suspended SU-8 microwires and bulk posts in various pyrolysis temperature conditions. [file 1556-276X-8-492-S1.docx]

**Supporting Information for the manuscript**

**“Monolithic carbon structures including suspended single nanowires and nanomeshes as a sensor platform”**

1. **Longitudinal tension and geometry of suspended carbon nanowires**

The longitudinal tension of the suspended carbon nanowire developed during pyrolysis was demonstrated by cutting the nanowire using focused ion-beam (FIB) milling. The carbon nanowire used in this experiment had dimensions (width and thickness of ~200 nm) similar to those of the carbon nanowires used in the experiments in the characterization of microstructures, thermal-electrical property, electrochemical behavior, and gas sensing capability. The ion beam was directed perpendicular to the wire length from the top side of the wire as described in figure S1(c). As the milling process preceded, the carbon nanowire bent upwards and it was finally cut off, as shown in figure S1 (d)-(g). This feature is indicative of the existence of a longitudinal tension gradient along the nanowire thickness. Negligible length reduction of the sectioned nanowires from the original nanowire before the FIB milling process indicates that the total amount of the longitudinal tension was not significant. The left sectioned nanowire was bent to a greater extent than the other sectioned nanowire. This is because of the difference in length between the two sectioned nanowires. The cutting position was 65.9 μm from the left carbon post and 21.3 μm from the right carbon post.

**2. Simulation of the diffusion-limited current of a suspended carbon nanowire**

The effect of nanowire geometries on the diffusion limited current was studied by numerically calculating the concentration profiles of redox species at the nanowires. The suspended carbon nanowires have high aspect ratio (>200) such that the effect of diffusion at the ends of the nanowire can be neglected. Therefore, a 2D model was used for the simulation. The diffusion equation in 2D is described as

$\frac{\partial c}{\partial t}=D\left(\frac{\partial^{2}c}{\partial x^{2}}+\frac{\partial^{2}c}{\partial y^{2}}\right)$ (1)

where *c* is the concentration and *D* is the diffusivity of [Fe(CN)_6_]^3-^/[Fe(CN)_6_]^4-^, and *x* and *y* are the coordinates of the system. It was assumed that no concentration gradient normal to the insulating substrate exists. The concentrations of the redox species along the simulation boundary were set to be the same as the concentrations used in bulk solution. Inward flux for oxidized species [Fe(CN)_6_]^3-^ and outward flux for reduced species [Fe(CN)_6_]^4-^ were determined according to Butler-Volmer kinetics [1]. The diffusion limited current was obtained following Fick’s first law.

**References**

1. Yang X, Zhang G: **The voltammetric performance of interdigitated electrodes with different electron-transfer rate constants**. *Sens Actuators B* 2007 **126:**624-631.


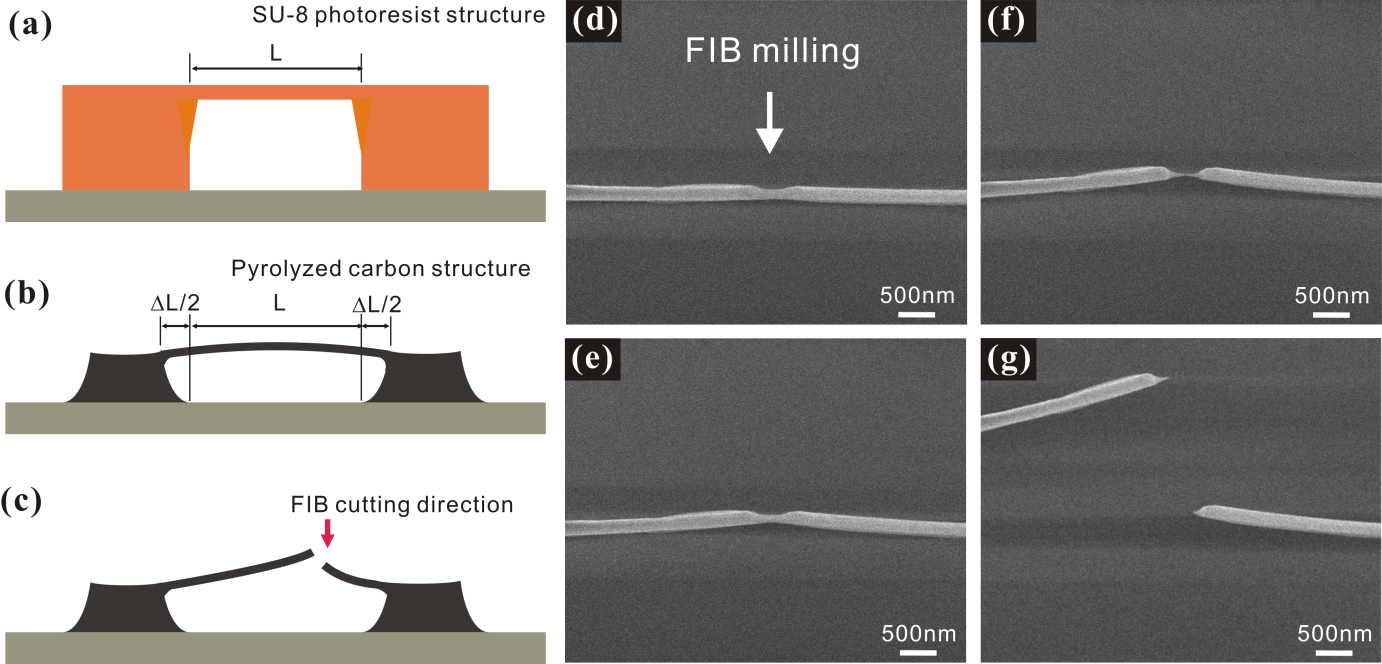


Figure S1 Schematic diagrams of (a) a SU-8 photoresist structure before pyrolysis and (b) a corresponding pyrolyzed carbon structure, (b) schematic diagram of a FIB processed suspended carbon nanowire, and (d)-(g) SEM images of a single suspended carbon nanowire as a FIB milling process proceeds.


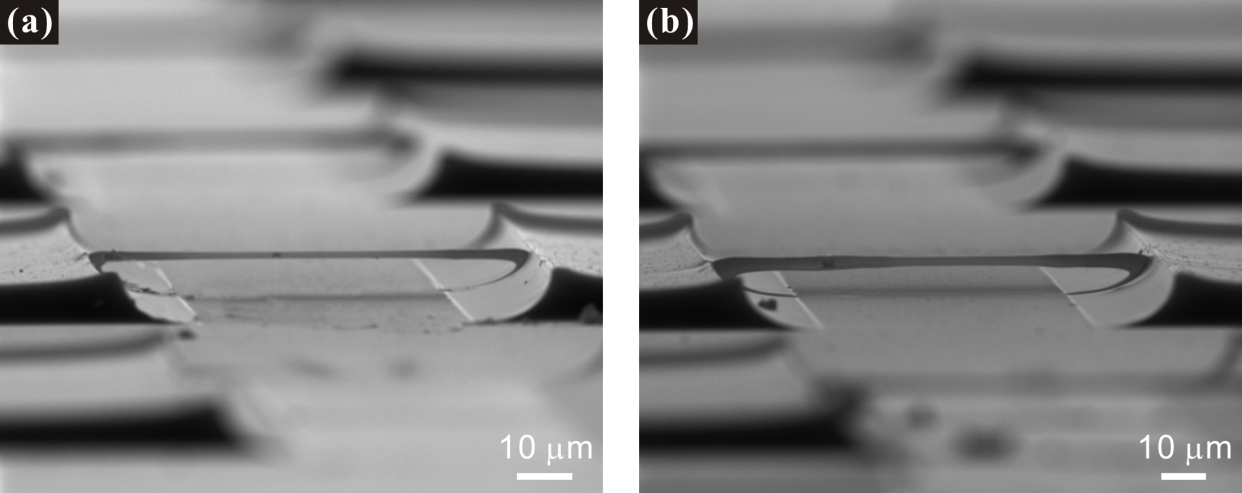
Figure S2 SEM images of bridge-shaped carbon nanowires with bent supports corresponding to the polymer suspended microwires of (a) 2 μm width and (b) 3 μm width as listed in Table S1.


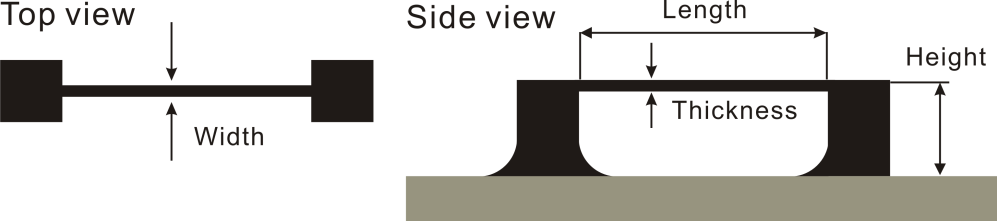
Table S1 Structural dimension change of suspended carbon nanostructures through the pyrolysis process

| Suspended polymer microstructure  (before pyrolysis) | | | | Suspended carbon nanostructures  (after pyrolysis) | | | |
| --- | --- | --- | --- | --- | --- | --- | --- |
| Width  [μm] | Thickness  [μm] | Height  [μm] | Length  [μm] | Width  [μm] | Thickness  [μm] | Height  [μm] | Length  [μm] |
| 1 | 2 | 27.3 | 54.0 | 0.195 | 0.21 | 11.4 | 89.4 |
| 2 | 6.5 |  |  | 0.6 | 1.56 |  |  |
| 3 | 7.3 |  |  | 0.74 | 1.99 |  |  |
| 4 | 10.2 |  |  | 1.12 | 2.87 |  |  |


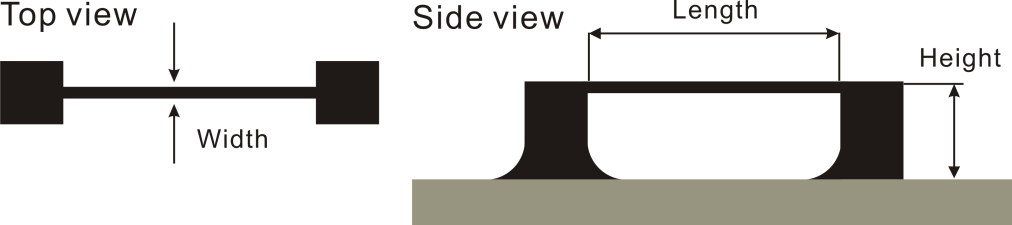
Table S2 Structural dimension changes of suspended SU-8 microwires and bulk posts in various pyloysis temperature conditions. All the temperature conditions include natural cooling down processes of the microwires after the pyrolysis processes listed in the table.

| Pyrolysis temperature  conditions | Suspended wire | | Bulk post |
| --- | --- | --- | --- |
|  | Width [μm] | Length [μm] | Height [μm] |
| Before pyrolysis | 1 | 55.8 | 24 |
| 300^o^C for 2 min | 0.675 | 63.5 | 20.2 |
| 350^o^C for 2 min | 0.495 | 79.2 | 15.3 |
| 400^o^C for 2 min | 0.430 | 82.7 | 12.9 |
| 450^o^C for 2 min | 0.359 | 84.4 | 11.5 |
| 500^o^C for 2 min | 0.305 | 85.0 | 11.4 |
| 500^o^C for 60 min | 0.296 | 85.8 | 10.6 |
| 350^o^C for 60 min  and 900^o^C for 60 min | 0.195 | 89.4 | 10.0 |
